# Standardized Tool for Prophylaxis (SToP) of venous thromboembolism: An asynchronous curriculum to enhance venous thromboembolism prevention in pediatric inflammatory bowel disease

**DOI:** 10.1002/jpr3.70281

**Published:** 2026-10-26

**Authors:** Hilary K. Michel, Arianna Henning, Jeff Barbee, Jaclyn Gish‐Lieberman, Sabina Ali

**Affiliations:** ^1^ Division of Gastroenterology Nationwide Children's Hospital Columbus Ohio USA; ^2^ Department of Pediatrics The Ohio State University College of Medicine Columbus Ohio USA; ^3^ The Office of Curriculum and Scholarship, The Ohio State University College of Medicine Columbus Ohio USA; ^4^ Ohio Department of Taxation Columbus Ohio USA; ^5^ UCSF Benioff Children's Hospital, University of California San Francisco San Francisco California USA

**Keywords:** continuing education, Crohn's disease, medical education, online curriculum, ulcerative colitis

## Abstract

**Objectives:**

Compared to those without inflammatory bowel disease (IBD), pediatric patients with IBD are at three‐ to sevenfold increased risk for venous thromboembolism (VTE), a complication associated with substantial morbidity and mortality. Despite this risk, gaps persist in clinician awareness, and standardized education and guidelines regarding prevention are lacking. We aimed to improve pediatric healthcare provider knowledge and confidence regarding VTE prevention in patients hospitalized with active IBD.

**Methods:**

We created a three‐module, online, asynchronous educational course. Using a pre–post survey design, we studied self‐reported confidence and knowledge. Responses were measured using 5‐point (confidence) and 3‐point (knowledge) Likert scales, with higher scores reflecting greater confidence/knowledge. Post‐course satisfaction was also measured. Change in confidence was analyzed using linear regression, and knowledge change using Wilcoxon signed‐rank tests.

**Results:**

Sixty‐five participants completed the course; 55% attending physicians, 71% female, 73% practiced in an academic setting. Mean (*M*) confidence in VTE prevention increased from pre‐ (*M* = 2.57, standard deviation [SD] = 0.98) to post‐test (*M* = 4.07, SD = 0.60), *p* < 0.001. Median (Md) knowledge also increased from the module one pre‐ (Md = 3.00, interquartile range [IQR] = 1.00) to post‐test (Md = 3.00, IQR = 0.00), *p* < 0.001, and module two pre‐ (Md = 2.00, IQR = 1.00) to post‐test (Md = 3.00, IQR = 0.00), *p* < 0.001. Participants reported high levels of satisfaction with the curriculum.

**Conclusions:**

A brief, asynchronous educational intervention significantly improved clinician knowledge and confidence in VTE prevention for pediatric patients hospitalized with active IBD. Scalable online curricula may represent an effective strategy to address educational gaps and support improved clinical care in high‐risk populations.

## Abstract (highlights)

What Is Known

- Pediatric patients hospitalized with active inflammatory bowel disease (IBD) have an increased risk of venous thromboembolism (VTE).
- In adults with IBD, inpatient VTE prophylaxis is standard practice and safely reduces VTE risk.
- However, no standard prophylaxis approach exists for hospitalized pediatric patients, partly due to limited provider knowledge and comfort.

What Is New

- We developed a brief, provider‐facing, asynchronous online curriculum on VTE prevention in hospitalized pediatric patients with active IBD.
- Pre‐ and post‐assessments showed significant improvements in provider knowledge and confidence regarding VTE prophylaxis, with high participant satisfaction.

## 1 INTRODUCTION

Venous thromboembolism (VTE) is a recognized complication in hospitalized pediatric patients with inflammatory bowel disease (IBD), associated with significant morbidity and mortality.^1^ Meta‐analysis data demonstrate that children and adolescents with IBD have an approximately three‐ to sevenfold higher risk of VTE compared to those without IBD, with the absolute risk being highest during hospitalization and around the time of diagnosis.^2^ While prophylactic measures, including pharmacologic interventions, have been shown to effectively prevent VTE formation, their implementation in pediatric IBD remains inconsistent. In adult IBD management, VTE prevention strategies are well established and considered standard of care. This is lacking in pediatrics.^3^ This gap is likely attributable to limited awareness of VTE and insufficient confidence in prescribing and monitoring prophylaxis medications.^4^ To address this unmet need, we developed an online educational module and toolkit to standardize and improve prevention for children and adolescents hospitalized with active IBD.

The specific aims of this project were as follows:

(1) To provide evidence‐based education to clinicians caring for hospitalized pediatric IBD patients regarding VTE risks and prophylaxis.
(2) To create a web‐based toolkit outlining the process of developing a standardized VTE risk screening and prophylaxis program at an IBD center.
(3) To develop patient‐facing educational materials regarding VTE risk assessment and prophylaxis in pediatric IBD patients for easy dissemination across IBD care centers.
(4) To evaluate the effectiveness of this evidence‐based education, as indicated by participant knowledge, confidence, and course satisfaction.

## 2 METHODS

### 2.1 Ethics statement

This project was reviewed by the Nationwide Children's Hospital (NCH) institutional review board and deemed Not Human Research (STUDY00004531: Standardized Tool for Prolphylaxis (SToP) of VTE).

### 2.2 Methods

#### 2.2.1 Curriculum development and content

As mentioned above, children hospitalized with active IBD are at high risk for serious complications related to VTE. However, universal guidelines are lacking for VTE prophylaxis in pediatric IBD, and pediatric providers continue to report variable practices and concerns regarding knowledge and confidence in safely prescribing prophylaxis.^4^

To address our specific aims, we developed asynchronous modules to increase healthcare providers' knowledge and confidence regarding the development and adoption of a VTE protocol for pediatric patients at their respective institutions. Asynchronous courses are attractive for their on‐demand availability and scalability compared to live educational opportunities either online or in person.^5^, ^6^ Practicing clinicians' time for professional development is severely limited when it must compete with patient care, administrative duties, and attending medical students and residents.^7^ In addition to convenience, the web‐based nature of asynchronous courses affords learning modalities beyond didactic lecture or textbooks, such as multimedia, authentic cases, and interaction, creating a more engaging and satisfying learner experience.^7^, ^8^

However, high attrition rates have also been associated with asynchronous healthcare learning modules due to lack of protected time or accountability compared with required lecture attendance.^7^, ^9^, ^10^ In other words, the anytime, anywhere flexibility of on‐demand modules can have the opposite and undesirable effect of falling to the bottom of the priority list after initial course registration. Some research suggests that brief on‐line modules can achieve high retention if they offer continuing medical education (CME),^9^, ^11^ sound instructional design,^9^, ^12^ and ready‐made resources to support buy‐in for new protocols at both the institution and patient level.^6^, ^10^

To create asynchronous modules that overcome attrition pitfalls, two principal investigators (H.K.M. and S.A.), both pediatric gastroenterologists, engaged instructional designers at a partnering college of medicine. The partnership combined subject matter expertise with platforms, tools, and design ability to create three robust, on‐demand modules. The instructional designers leveraged the three steps of Backward Design^12^, ^13^ for course development: identify the desired learning outcomes, determine acceptable evidence, and plan learning experiences.

The principal investigators identified four learning outcomes with the aim of VTE protocol adoption through increased knowledge and confidence. (1) Describe the pathophysiology of VTE in IBD. (2) Prevent VTE through screening for VTE risk, administering VTE prophylaxis, and monitoring to ensure safety. (3) Develop a standardized VTE risk screening and prevention protocol. (4) Educate patients and families about VTE risk and prophylaxis. Evidence of learning was supported through module quizzes to evaluate participant knowledge. Learning experience design centered on animated videos that link protocols to an authentic patient case and downloadable, one‐page handouts for easy distribution to healthcare providers and patient families.

The curriculum consists of three modules focused on VTE prevention in pediatric patients hospitalized with active IBD: the pathophysiology of VTE in pediatric IBD, VTE risk assessment and safe prophylaxis practices, and application of quality improvement methodology to support local development and implementation of VTE screening and prevention protocols personalized to a center's needs and resources. Each module presents didactic information, including key terminology and explanations of complex processes through text, graphics, and videos in a user‐friendly and accessible format. Module 2 introduces Alex, a fictional 16‐year‐old girl with ulcerative colitis, through two case videos. Through the first video, learners see—not just read about—how proper screening for VTE risk impacts the decision to start pharmacological VTE prophylaxis. A provider‐facing handout (Figure 1) follows the video, capturing the key points of VTE risk factors and prevention. The second video continues with Alex, demonstrating how to monitor pharmacologic and non‐pharmacologic VTE therapies. A handout containing a sample VTE prevention protocol follows (Figure S1). A final handout, accessed in the third module, is designed for patients and caregivers and presents IBD blood clot prevention information in language accessible to non‐medical professionals (Figure S2).

**Figure 1:**
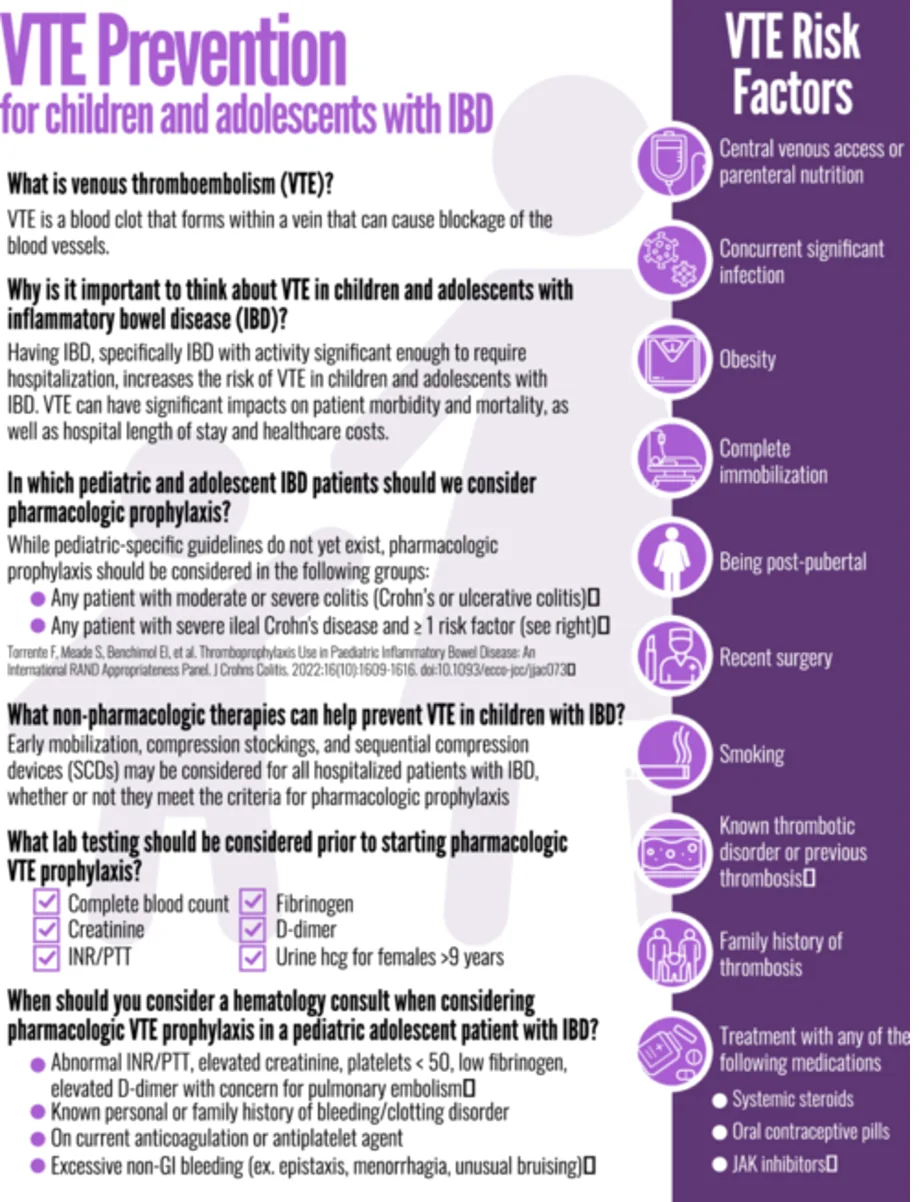
VTE Prevention for children and adolescents with IBD provider‐facing handout. GI, gastrointestinal; IBD, inflammatory bowel disease; INR, international normalized ratio; PTT, partial thromboplastin time; SCD, sickle cell disease; VTE, venous thromboembolism.

The SToP VTE course is accessible through a learning management system (LMS) curated by the partnering university for on‐demand professional continuing education opportunities. Due to grant support from the North American Society for Pediatric Gastroenterology, Hepatology and Nutrition (NASPGHAN) Foundation, it was available for free to NASPGHAN members for a 2‐year period and is now available for a small fee that supports LMS upkeep. Prior to widespread dissemination, the course was reviewed and pilot tested by a group of pediatric experts including pediatric hematologists, gastroenterologists with expertise in IBD and VTE‐prevention, and clinical pharmacists. Based on this pilot testing, we determined the entire course could be completed in approximately 1 h.

#### 2.2.2 Study design and evaluation strategy

This was a single‐arm, pre–post study of pediatric healthcare providers. This design allowed us to examine changes in participants' VTE confidence and knowledge as a result of the course. Questions addressing participant confidence using VTE prophylaxis were completed pre‐course and then repeated post‐course, while module‐specific pre‐ and post‐tests evaluated knowledge acquisition. Confidence items were measured on a 1–5 Likert‐type scale with higher values indicating greater confidence. Knowledge scores ranged from 0 to 3, with higher values indicating greater knowledge. Post‐course satisfaction was also measured on a 5‐point Likert‐type scale with higher values indicating greater satisfaction (Table S1, Survey Questions).

#### 2.2.3 Dissemination

Providers were recruited via various pediatric gastroenterology (GI) society communications and listservs (i.e., NASPGHAN, ImproveCareNow, PEDGI [Pediatric GI Bulletin Board] Listserv) between 2025 and 2026. Approximately 10 separate invitations were sent over a period of 6 months.

### 2.3 Statistical analysis

Descriptive statistics using *N*s and percents were used to summarize study participants and post‐test program satisfaction. Means and standard deviations and medians and interquartile ranges (IQRs) were used, as appropriate, to describe pre‐ and post‐curriculum confidence and knowledge.

Cronbach's *α* was calculated to determine whether items for the confidence scale had adequate internal consistency reliability at pre‐test (*α* = 0.83) and post‐test (*α* = 0.84).

A linear regression was conducted to evaluate whether mean changes in confidence were statistically significant. Composite scores were created to assess mean differences in confidence from pre‐test to post‐test. A mean difference score (post − pre) was modeled as the dependent variable in the linear regression predicting the mean change (equivalent to a paired‐samples *t* test). Parameter estimates were pooled across imputations according to Rubin's rules. Visual inspection of difference score histograms and *Q*–*Q* plots suggested that data were normally distributed. Furthermore, skewness and kurtosis of the difference scores were within acceptable limits (skewness = 0.45, kurtosis = −0.55).

Due to violations of normality assumptions, a Wilcoxon signed‐rank test was conducted to assess whether changes in knowledge in modules one and two were statistically significant.

Multiple imputation (*m* = 5) using multivariate imputation by chained equations (mice) was used to account for missing data in the post‐survey data.

All analyses were conducted in RStudio (R Core Team, 2023).

## 3 RESULTS

### 3.1 Participant demographics

Participants (*N* = 65) were 71% female, 52% White and 48% non‐White, including Asian (29%), Black/African American (3%), multiracial (3%), those who selected other (3%), and those who preferred not to answer (10%). Most participants were attending physicians (55%) and reported practicing in an academic setting (73%). Most participants reported being between 31 and 40 years old (52%) and had been practicing for 5 years or less (55%) (Table 1).

**Table 1:** Participant demographic information.

Characteristic	*N* (%)
Gender	
Female	45 (71)
Male	17 (27)
Transgender female	1 (2)
Race	
Caucasian/White	33 (52)
Asian	18 (29)
Prefer not to say	6 (10)
Black/African American	2 (3)
Other	2 (3)
Multiracial	2 (3)
Ethnicity	
Non‐Hispanic	50 (78)
Prefer not to say	8 (13)
Hispanic	6 (9)
Role	
Attending pediatric gastroenterologist	35 (55)
Fellow	19 (30)
Advanced practice provider	4 (6)
Pharmacist	2 (3)
Other	2 (3)
Nurse	1 (2)
Resident	1 (2)
Practice setting	
Academic	47 (73)
Large health system	12 (19)
Private	4 (6)
Other	1 (2)
Age (years)	
20–30	6 (9)
31–40	33 (52)
41–50	14 (22)
50+	11 (17)
Years practicing in role	
0–5	35 (55)
6–10	15 (23)
11–15	5 (8)
16–20	3 (5)
>20	6 (9)

### 3.2 Pre–post VTE confidence and knowledge scores

Participants' confidence and knowledge increased significantly from pre‐test to post‐test (Table 2). Linear regression revealed that participants' mean (*M*) confidence in VTE prevention increased significantly from pre‐ (*M* = 2.57, SD = 0.98) to post‐test (*M* = 4.07, SD = 0.60), *b* = 1.50, *p* < 0.001. Wilcoxon signed rank tests revealed a significant increase in participants' median (Md) knowledge from the module one pre‐test (Md = 3.00, IQR = 1.00) to module one post‐test (Md = 3.00, IQR = 0.00), *V* = 289.50, *p* < 0.001, *r* = 0.63, and module two pre‐test (Md = 2.00, IQR = 1.00) to module two post‐test (Md = 3.00, IQR = 0.00), *V* = 490.00, *p* < 0.001, *r* = 0.32. These effect sizes are large and medium, respectively. Finally, there were high levels of satisfaction with the course amongst those who completed the post‐course satisfaction survey (*N* = 39), with nearly all noting that it was brief, engaging, and that they would recommend it to colleagues (Table 3).

**Table 2:** Pre–post confidence and knowledge scores.

Scale	Pre‐test mean (SD)	Post‐test mean (SD)	Pre‐test median (IQR)	Post‐test median (IQR)
Confidence (*n* = 65)	2.57 (0.98)	4.07 (0.60)	2.60 (1.20)	4.08 (0.72)
Module 1 Knowledge (*n* = 64)	2.55 (0.67)	2.96 (0.19)	3.00 (1.00)	3.00 (0.00)
Module 2 Knowledge (*n* = 55)	2.26 (0.76)	2.93 (0.34)	2.00 (1.00)	3.00 (0.00)

*Note*: Confidence scores ranged from 1 to 5, and knowledge scores ranged from 0 to 3, with higher scores indicating greater levels of confidence and knowledge, respectively.

Abbreviations: IQR, interquartile range; SD, standard deviation.

**Table 3:** Curriculum satisfaction survey results.

Items	Mean (SD)	Median (IQR)
This curriculum improved my knowledge about VTE risk in hospitalized youth with IBD	4.31 (0.69)	4.00 (1.00)
This curriculum improved my skills related to screening for VTE risk	4.35 (0.54)	4.00 (1.00)
This curriculum improved my knowledge about prescribing prophylaxis for hospitalized youth with IBD	4.31 (0.66)	4.00 (1.00)
I found the course content to be engaging	4.28 (0.72)	4.00 (1.00)
I would recommend this content to a colleague	4.44 (0.64)	5.00 (1.00)
This course met my expectations	4.31 (0.69)	4.00 (1.00)

*Note*: Satisfaction scores ranged from 1 to 5, with higher scores indicating greater levels of satisfaction.

Abbreviations: IBD, inflammatory bowel disease; IQR, interquartile range; SD, standard deviation; VTE, venous thromboembolism.

## 4 DISCUSSION

Using a brief, asynchronous, online curriculum for pediatric clinicians on VTE prevention in hospitalized pediatric patients with active IBD, we significantly improved participants' comfort and knowledge, with high levels of curricular satisfaction. This curriculum addresses an important and under‐recognized patient safety issue in pediatric IBD care, intervening upon which could have marked impacts on patient morbidity and mortality.

Several features may have contributed to the curriculum's effectiveness, including multidisciplinary expert input, evidence‐based content, and use of asynchronous learning strategies designed for busy clinicians.^5^, ^8^, ^10^ Downloadable provider‐ and patient‐facing handouts also allowed for reinforcement of educational content after completion of the modules. The modules were also designed to guide the learner through content in a logical manner, starting with baseline knowledge in modules one and two and then transitioning to content on use of quality improvement methodology to develop and implement a VTE prophylaxis plan locally. The educational intervention was intentionally designed to be broadly applicable across diverse pediatric care settings, including academic medical centers, community hospitals, and mixed adult–pediatric environments. The content focuses on core principles of VTE risk assessment, safe prophylaxis, and quality improvement methodology rather than institution‐specific protocols, allowing practices to adapt the material to local workflows, resources, and patient populations. Targeted dissemination through professional societies and learning networks also helped increase visibility and engagement. Study participants represented a range of professional roles and practice settings, suggesting relevance and accessibility across institutions.

Finally, participants showed significant improvements in both knowledge and self‐reported confidence related to VTE prevention with large effect sizes, suggesting the curriculum was both effective and clinically relevant. Our findings are consistent with prior studies demonstrating that asynchronous, online curricula can improve learner knowledge in an accessible, highly satisfactory fashion.^5^, ^6^, ^8^, ^9^, ^10^, ^11^

This study's single‐arm pre–post design is an important limitation. Without a control group, improvements in knowledge and confidence cannot be attributed exclusively to the curriculum, as external educational experiences or testing effects may have contributed to observed changes. Future studies should evaluate the curriculum using controlled or randomized designs. Selection bias may also have influenced our findings. Participation was voluntary, and recruitment occurred through professional society communications and listservs, which may have preferentially attracted clinicians already interested in VTE prevention or pediatric IBD. Furthermore, only a subset of participants completed post‐course satisfaction surveys, which may have contributed to response bias and inflated satisfaction estimates. Finally, generalizability may also be limited because most participants were attending physicians practicing in academic centers. The effectiveness and perceived value of the curriculum may differ among clinicians practicing in community hospitals, mixed adult‐pediatric settings, or other healthcare environments.

Although the curriculum significantly improved clinician knowledge and confidence, important intermediary outcomes, this study did not evaluate whether these gains translated into changes in clinical practice or patient outcomes, such as increased VTE risk assessment, appropriate prophylaxis prescribing, implementation of standardized institutional protocols, reductions in VTE events, or improvements in patient safety. Future studies should focus on determining whether this educational intervention leads to sustained practice change and measurable improvements in the quality and safety of care for hospitalized pediatric patients with IBD.

## 5 CONCLUSION

We developed and disseminated a structured, online, asynchronous educational program to improve clinician knowledge and confidence in preventing VTE in youth hospitalized with active IBD. The three‐module course addressed VTE pathophysiology, risk assessment and safe prophylaxis, and application of quality improvement methodology to support local implementation. Our results demonstrate significant improvements in both self‐reported confidence and knowledge, with large effect sizes. The program was successfully disseminated through national professional networks and was designed to be scalable, sustainable, and adaptable across diverse practice settings. These findings suggest that brief, asynchronous educational interventions can effectively address important patient safety gaps and support practice improvement in pediatric subspecialty care.

## CONFLICT OF INTEREST STATEMENT

The authors declare no conflicts of interest.

## Supporting information

Supplemental Figure 1. VTE Prevention Protocol for children and adolescnes with IBD Provider‐Facing Handoung.

Supplemental Figure 2. Inflammatory Bowel disease blot clot prevention information for patients and families.

Supplemental Table 1. Survey Questions.

## Data Availability

The data that support the findings of this study are available from the corresponding author upon reasonable request.
