# Supplementary material for: Standardized Tool for Prophylaxis (SToP) of venous thromboembolism: An asynchronous curriculum to enhance venous thromboembolism prevention in pediatric inflammatory bowel disease
Source: JPGN Rep. 2026 Oct 26:10.1002/jpr3.70281. Online ahead of print. doi: 10.1002/jpr3.70281 (PMC13710940; doi:10.1002/jpr3.70281)
Supplement: Supplementary file 1 [file JPR3-9999-0-s002.pdf]

VTE Prevention Protocol

for children and adolescents with IBD

There are not yet universal guidelines for venous thromboembolism (VTE) prophylaxis in pediatric patients with inflammatory bowel disease (IBD) admitted to the hospital. Use this example as a starting point as you build a protocol at your center.

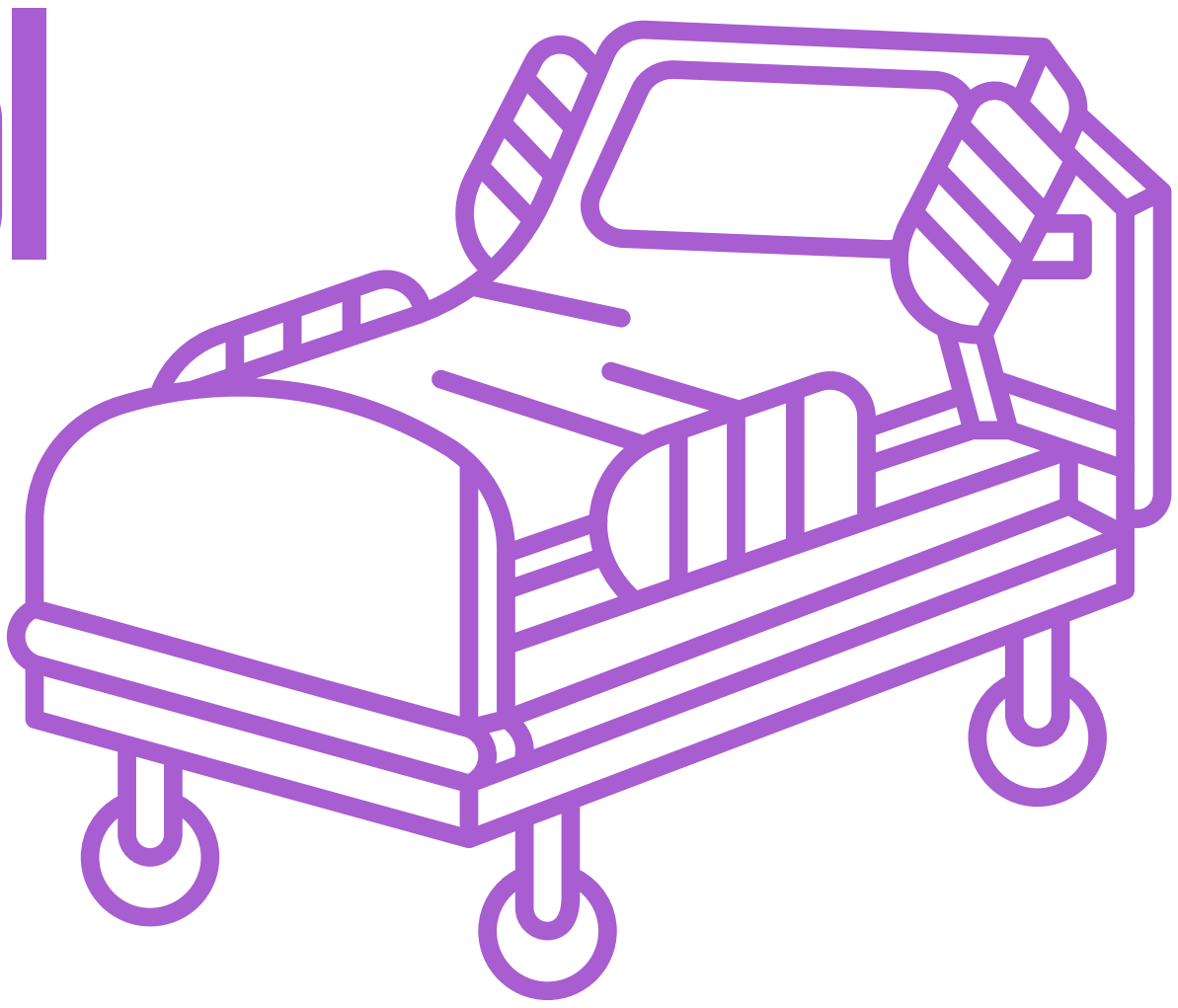

Starting dose

Enoxaparin 0.5 mg/kg/dose (max 30 mg/dose) subcutaneous every 12 hours
Or 1 mg/kg/dose (max 30 mg/dose) subcutaneous every 24 hours

Monitoring

Draw anti-Xa level 4 hours after 3rd or 4th dose, whichever occurs during the daytime
Goal level: 0.1-0.3 units/mL, leave does as it

- If level < 0.1, increase dose by 15%
- If level 0.3-0.5, decrease dose by 15%
- If level > 0.5, consult hematology for assistance in dosing

Recheck

After any dose adjustment, recheck anti-Xa level 4hours after 2nd dose until goal is reached

Guidance for Holding and Discontinuing Enoxaparin

- Hold morning dose on the day of endoscopy
- Hold 2 doses before any surgery
- Continue enoxaparin until hospital discharge

Practical Notes and Caveats

- Preferred enoxaparin dosing may differ slightly between centers
- Some centers choose not to check anti-Xa levels when enoxaparin is being used for VTE prevention
- Some centers require hematology or surgery approval prior to starting enoxaparin
- In patients <40 kg or >100 kg dosing may need to be discussed
- Dose adjustment is required for renal insufficiency
