# Supplementary material for: Standardized Tool for Prophylaxis (SToP) of venous thromboembolism: An asynchronous curriculum to enhance venous thromboembolism prevention in pediatric inflammatory bowel disease
Source: JPGN Rep. 2026 Oct 26:10.1002/jpr3.70281. Online ahead of print. doi: 10.1002/jpr3.70281 (PMC13710940; doi:10.1002/jpr3.70281)
Supplement: Supplementary file 2 [file JPR3-9999-0-s003.pdf]

INFLAMMATORY BOWEL DISEASE

blood clot prevention information for patients and families

A blood clot is like a group of sticky blood cells. It usually forms to stop bleeding after a cut or injury. But sometimes, a clot can block a tiny tube in your body (called a vein) and disrupt normal blood flow. Doctors call this a venous thrombosis (VT). If the clot moves to another part of your body, it's called a venous thromboembolism (VTE). VTs and VTEs are a problem because they hinder blood flow.

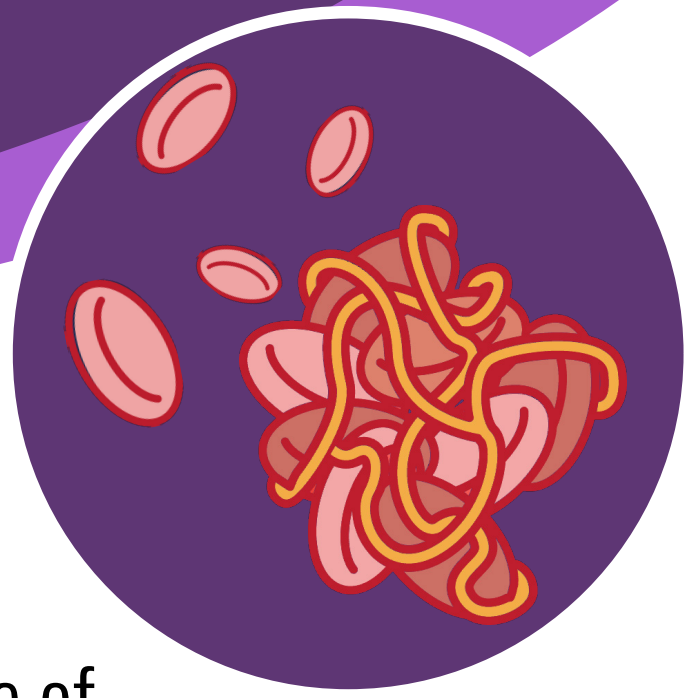

BLOOD CLOTS AND PATIENTS WITH IBD

People with IBD, like Crohn's disease and ulcerative colitis, have a higher chance of getting blood clots, especially during active inflammation. Some other factors, like use of steroids and lack of movement - can also make this risk go up.

BLOOD CLOT SYMPTOMS

Symptoms depend on where the blood clot is in the body. If they are in the veins of the arms or legs, they can cause swelling, redness, or pain in that limb.

BLOOD CLOT PREVENTION

The good news is that many blood clots are preventable with:

- ★ Movement
- ★ Compression Socks & Sequential Compression Devices
- ★ Blood thinners (E.g., Enoxaparin)

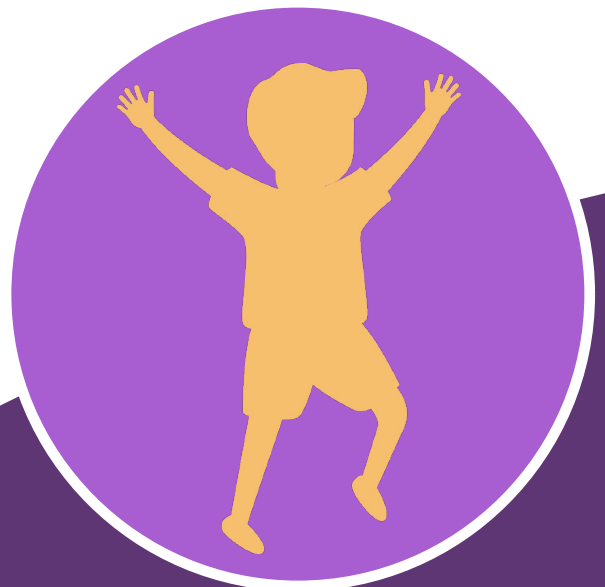

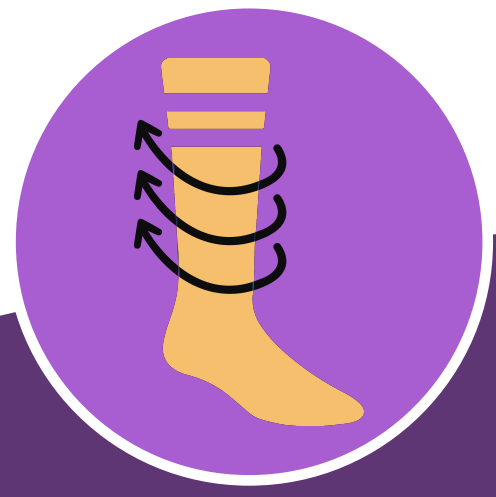

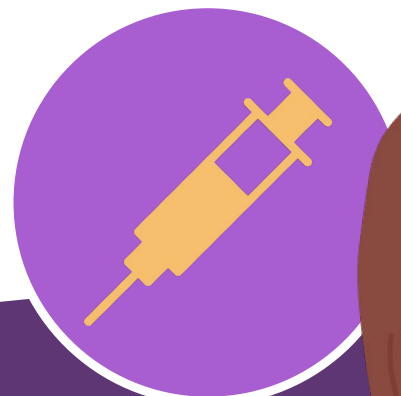

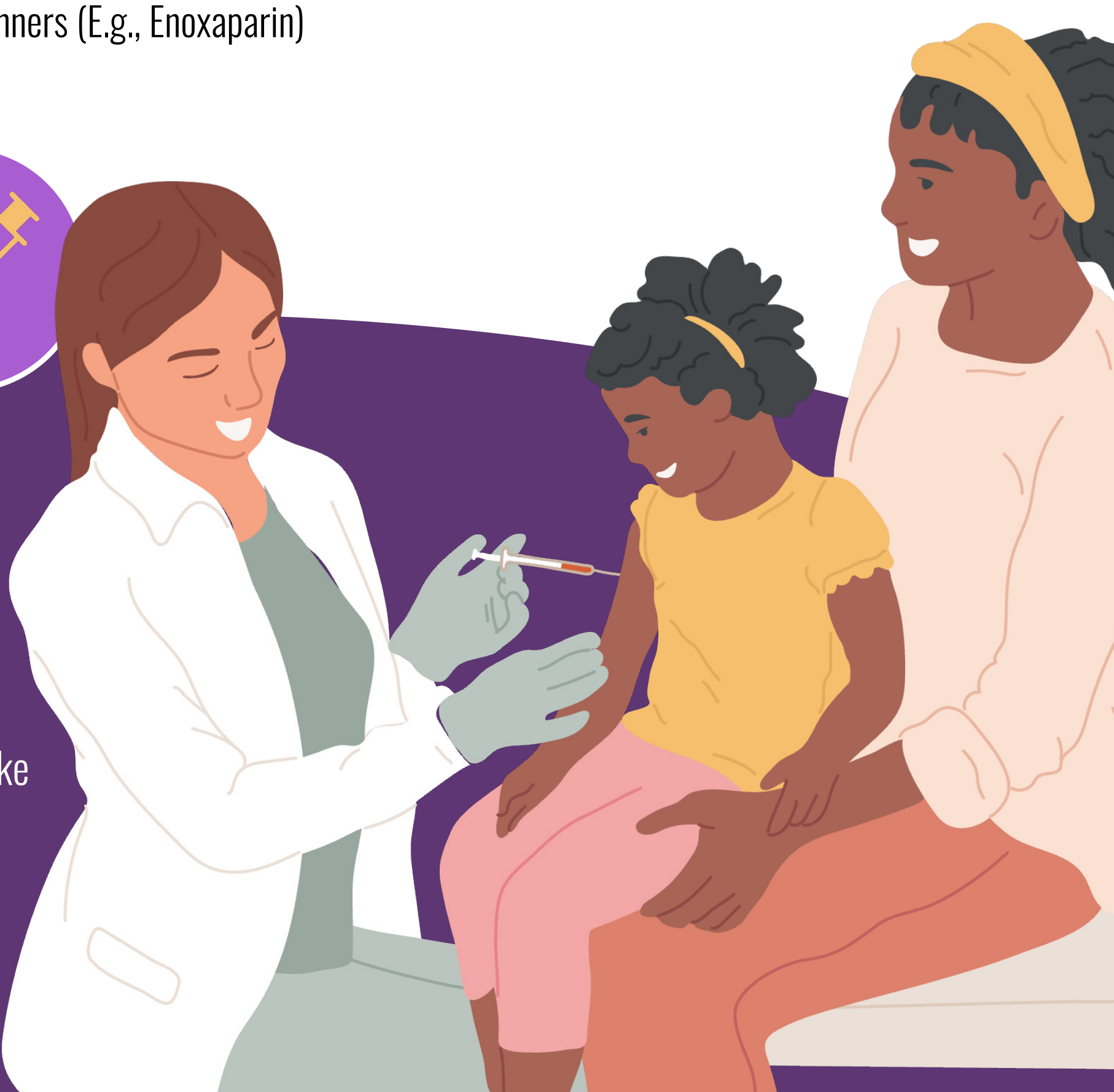

MAKE INJECTIONS MORE PLEASANT

Talk with your healthcare team about different ways to make injections more pleasant for your child, such as:

- Distraction (video, music, etc.)
- Diaphragmatic Breathing
- Numbing Cream
- Child Life Specialist
