# Supplementary material for: Standardized Tool for Prophylaxis (SToP) of venous thromboembolism: An asynchronous curriculum to enhance venous thromboembolism prevention in pediatric inflammatory bowel disease
Source: JPGN Rep. 2026 Oct 26:10.1002/jpr3.70281. Online ahead of print. doi: 10.1002/jpr3.70281 (PMC13710940; doi:10.1002/jpr3.70281)
Supplement: Supplementary file 3 [file JPR3-9999-0-s001.docx]

**Supplementary Table 1. Survey Questions**

1. ***Confidence Items***

Items

Ordering non-medication venous thromboembolism (VTE) prophylaxis for youth hospitalized with active inflammatory bowel disease (IBD) (i.e., early ambulation, sequential compression devices, compression stockings).

Ordering medication VTE prophylaxis (i.e., enoxaparin) for youth hospitalized with active IBD.

Monitoring patients receiving medication VTE prophylaxis (i.e., enoxaparin) for youth hospitalized with active IBD.

Working with my team locally to develop a VTE prophylaxis protocol for youth hospitalized with active IBD.

Educating patients and caregivers about the risk of VTE in youth hospitalized with active IBD.

| 2. ***Module One Knowledge Assessment Items*** | |
| --- | --- |
| Item | Answer Options |
| Deep vein thromboses (DVTs) most commonly occur in which area of the body? | a) Arms b) Legs c) Liver d) Pelvis |
| During an IBD flare, which of the following factors is decreased, contributing to hypercoagulability? | a) Factor VIII b) Protein S c) Platelets d) Fibrinogen |
| Virchow’s Triad includes which of the following components? | a) Anemia b) Vessel wall injury c) Electrolyte imbalance d) Bleeding |
| 3. ***Module Two Knowledge Assessment Items*** | |
| Item | Answer Options |
| All the following increase a patient's risk of VTE **except**: | a) Post-pubertal stage b) Perianal Crohn’s disease c) Oral contraceptive pills d) Central venous access e) Obesity |
| Hematology consultation for admitted patients with active IBD should be considered in all the following situations **except**: | a) Severe colitis with a hemoglobin of 8 b) Personal history of thrombosis c) Abnormal coagulation factors at baseline d) Family history of thrombotic disorder e) Excessive non-gastrointestinal bleeding (i.e., epistaxis, menorrhagia) |
| Which of the following may help prevent venous thromboembolic events in hospitalized patients with IBD? | a) Pharmacologic prophylaxis (I.e. low molecular weight heparin) b) Compression stockings c) Early ambulation d) Sequential compression devices (SCDs) e) All of the above |

4. ***Curriculum Satisfaction Items***

Items

This curriculum improved my knowledge about VTE risk in hospitalized youth with IBD.

This curriculum improved my skills related to screening for VTE risk.

This curriculum improved my knowledge about prescribing prophylaxis for hospitalized youth with IBD.

I found the course content to be engaging.

I would recommend this content to a colleague.

This course met my expectations.
